# Mortality following elective abdominal aortic aneurysm repair in women

**DOI:** 10.1093/bjs/znab465

**Published:** 2022-03-03

**Authors:** V. N. Tedjawirja, A. J. Alberga, M. H. P. Hof, A. C. Vahl, M. J. W. Koelemay, R. Balm, in collaboration with the Dutch Society of Vascular Surgery, P.J. van den Akker, G.J. Akkersdijk, G.P. Akkersdijk, W.L. Akkersdijk, M.G. van Andringa de Kempenaer, C.H.P. Arts, J.A.M. Avontuur, O.J. Bakker, R. Balm, W.B. Barendregt, J.A. Bekken, M.H. Bender, B.L. Bendermacher, M. van den Berg, P. Berger, R.J. Beuk, J.D. Blankensteijn, R.J. Bleker, J.J. Blok, A.S. Bode, M.E. Bodegom, K.E. van der Bogt, A.P.M. Boll, M.H. Booster, B.L. Borger van der Burg, G.J. de Borst, W.T.G.J. Bos- van Rossum, J. Bosma, J.M.J. Botman, L.H. Bouwman, V. Brehm, M.T. de Bruijn, J.L. de Bruin, P. Brummel, J.P. van Brussel, S.E. Buijk, M.A. Buijs, M.G. Buimer, D.H. Burger, H.C. Buscher, E. Cancrinus, P.H. Castenmiller, G. Cazander, A.M. Coester, P.H. Cuypers, J.H. Daemen, I. Dawson, J.E. Dierikx, M.L. Dijkstra, J. Diks, M.K. Dinkelman, M. Dirven, D.E. Dolmans, R.C. van Doorn, L.M. van Dortmont, J.W. Drouven, M.M. van der Eb, D. Eefting, G.J. van Eijck, J.W. Elshof, B.H. Elsman, A. van der Elst, M.I. van Engeland, R.G. van Eps, M.J. Faber, W.M. de Fijter, B. Fioole, T.M. Fokkema, F.A. Frans, W.M. Fritschy, P.H. Fung Kon Jin, R.H. Geelkerken, W.B. van Gent, G.J. Glade, B. Govaert, R.P. Groenendijk, H.G. de Groot, R.F. van den Haak, E.F. de Haan, G.F. Hajer, J.F. Hamming, E.S. van Hattum, C.E. Hazenberg, P.P. Hedeman Joosten, J.N. Helleman, L.G. van der Hem, J.M. Hendriks, J.A. van Herwaarden, J.M. Heyligers, J.W. Hinnen, R.J. Hissink, G.H. Ho, P.T. den Hoed, M.T. Hoedt, F. van Hoek, R. Hoencamp, W.H. Hoffmann, W. Hogendoorn, A.W. Hoksbergen, E.J. Hollander, M. Hommes, C.J. Hopmans, L.C. Huisman, R.G. Hulsebos, K.M. Huntjens, M.M. Idu, M.J. Jacobs, M.F. van der Jagt, J.R. Jansbeken, R.J. Janssen, H.H. Jiang, S.C. de Jong, T.A. Jongbloed-Winkel, V. Jongkind, M.R. Kapma, B.P. Keller, A. Khodadade Jahrome, J.K. Kievit, P.L. Klemm, P. Klinkert, N.A. Koedam, M.J. Koelemaij, J.L. Kolkert, G.G. Koning, O.H. Koning, R. Konings, A.G. Krasznai, R.M. Krol, R.H. Kropman, R.R. Kruse, L. van der Laan, M.J. van der Laan, J.H. van Laanen, G.W. van Lammeren, D.A. Lamprou, J.H. Lardenoye, G.J. Lauret, B.J. Leenders, D.A. Legemate, V.J. Leijdekkers, M.S. Lemson, M.M. Lensvelt, M.A. Lijkwan, R.C. Lind, F.T. van der Linden, P.F. Liqui Lung, M.J. Loos, M.C. Loubert, K.M. van de Luijtgaarden, D.E. Mahmoud, C.G. Manshanden, E.C. Mattens, R. Meerwaldt, B.M. Mees, G.C. von Meijenfeldt, T.P. Menting, R. Metz, R.C. Minnee, J.C. de Mol van Otterloo, M.J. Molegraaf, Y.C. Montauban van Swijndregt, M.J. Morak, R.H. van de Mortel, W. Mulder, S.K. Nagesser, C.C. Naves, J.H. Nederhoed, A.M. Nevenzel-Putters, A.J. de Nie, D.H. Nieuwenhuis, J. Nieuwenhuizen, R.C. van Nieuwenhuizen, D. Nio, V.J. Noyez, A.P. Oomen, B.I. Oranen, J. Oskam, H.W. Palamba, A.G. Peppelenbosch, A.S. van Petersen, B.J. Petri, M.E. Pierie, A.J. Ploeg, R.A. Pol, E.D. Ponfoort, I.C. Post, P.P. Poyck, A. Prent, S. ten Raa, J.T. Raymakers, M. Reichart, B.L. Reichmann, M.M. Reijnen, J.A. de Ridder, A. Rijbroek, M.J. van Rijn, R.A. de Roo, E.V. Rouwet, B.R. Saleem, P.B. Salemans, M.R. van Sambeek, M.G. Samyn, H.P. van ’t Sant, J. van Schaik, P.M. van Schaik, D.M. Scharn, M.R. Scheltinga, A. Schepers, P.M. Schlejen, F.J. Schlosser, F.P. Schol, V.P. Scholtes, O. Schouten, M.A. Schreve, G.W. Schurink, C.J. Sikkink, A. te Slaa, H.J. Smeets, L. Smeets, R.R. Smeets, A.A. de Smet, P.C. Smit, T.M. Smits, M.G. Snoeijs, A.O. Sondakh, M.J. Speijers, T.J. van der Steenhoven, S.M. van Sterkenburg, D.A. Stigter, R.A. Stokmans, R.P. Strating, G.N. Stultiëns, J.E. Sybrandy, J.A. Teijink, B.J. Telgenkamp, M. Teraa, M.J. Testroote, T. Tha-In, R.M. The, W.J. Thijsse, I. Thomassen, I.F. Tielliu, R.B. van Tongeren, R.J. Toorop, E. Tournoij, M. Truijers, K. Türkcan, R.P. Tutein Nolthenius, Ç. Ünlü, R.H. Vaes, A.A. Vafi, A.C. Vahl, E.J. Veen, H.T. Veger, M.G. Veldman, S. Velthuis, H.J. Verhagen, B.A. Verhoeven, C.F. Vermeulen, E.G. Vermeulen, B.P. Vierhout, R.J. van der Vijver-Coppen, M.J. Visser, J.A. van der Vliet, C.J. Vlijmen - van Keulen, R. Voorhoeve, J.R. van der Vorst, A.W. Vos, B. de Vos, C.G. Vos, G.A. Vos, M.T. Voute, B.H. Vriens, P.W. Vriens, A.C. de Vries, D.K. de Vries, J.P. de Vries, M. de Vries, C. van der Waal, E.J. Waasdorp, B.M. Wallis de Vries, L.A. van Walraven, J.L. van Wanroij, M.C. Warlé, W. van de Water, V. van Weel, A.M. van Well, G.M. Welten, R.J. Welten, J.J. Wever, A.M. Wiersema, O.R. Wikkeling, W.I. Willaert, J. Wille, M.C. Willems, E.M. Willigendael, E.D. Wilschut, W. Wisselink, M.E. Witte, C.H. Wittens, C.Y. Wong, R. Wouda, O. Yazar, K.K. Yeung, C.J. Zeebregts, M.L. van Zeeland

**Affiliations:** 1 Department of Surgery, Amsterdam Cardiovascular Sciences, Amsterdam UMC, University of Amsterdam, Amsterdam, the Netherlands; 2 Department of Vascular Surgery, Erasmus University Medical Centre, Rotterdam, the Netherlands; 3 Dutch Institute of Clinical Auditing, Scientific Bureau, Leiden, the Netherlands; 4 Department of Epidemiology and Data Science, Amsterdam Public Health, Amsterdam UMC, University of Amsterdam, Amsterdam, the Netherlands; 5 Department of Surgery, OLVG, Amsterdam, the Netherlands

## Abstract

**Background:**

Previous studies have focused on patient-related risk factors to explain the higher mortality risk in women undergoing elective abdominal aortic aneurysm (AAA) repair. The aim of this study was to evaluate whether hospital-related factors influence outcomes following AAA repair in women.

**Methods:**

Patients undergoing elective AAA repair in 61 hospitals in the Netherlands were identified from the Dutch Surgical Aneurysm Audit registry (2013–2018). A mixed-effects logistic regression analysis was conducted to assess the effect of sex on in-hospital and/or 30-day mortality. This analysis accounted for possible correlation of outcomes among patients who were treated in the same hospital, by adding a hospital-specific random effect to the statistical model. The analysis adjusted for patient-related risk factors and hospital volume of open surgical repair (OSR) and endovascular aneurysm repair (EVAR).

**Results:**

Some 12 034 patients were included in the analysis. The mortality rate was higher in women than among men: 53 of 1780 (3.0 per cent) *versus* 152 of 10 254 (1.5 per cent) respectively. Female sex was significantly associated with mortality after correction for patient- and hospital-related factors (odds ratio 1.68, 95 per cent c.i. 1.20 to 2.37). OSR volume was associated with lower mortality (OR 0.91 (0.85 to 0.95) per 10-procedure increase) whereas no such relationship was identified with EVAR volume (OR 1.03 (1.01 to 1.05) per 10-procedure increase).

**Conclusion:**

Women are at higher risk of death after abdominal aortic aneurysm repair irrespective of patient- and hospital-related factors.

## Teaser

This study investigated whether women still have a higher mortality risk than men following elective repair of abdominal aortic aneurysms, after accounting for both patient- and hospital-related factors. It was found that female sex remained associated with mortality.

## Introduction

Patients with an abdominal aortic aneurysm (AAA) can be treated electively by open surgical repair (OSR) or endovascular aneurysm repair (EVAR)^1^. Previous studies^2–5^ have shown that excess perioperative mortality is evident among women following both types of repair. Well known patient-related risk factors are associated with increased mortality risk, including age, cardiac and pulmonary co-morbidity, and impaired renal function^6–9^. Despite correction for such factors, female sex has persistently been associated with increased mortality^2,8^.

Hospital-level factors such as expertise in AAA surgery may influence patient outcomes. Volume can be used as a proxy for expertise and has been found to have an inverse relationship with mortality^1,10,11^. However, previous studies^2,8,12^ have focused only on patient-related factors. The aim of this study was to establish whether hospital-level factors could explain some of the differences in outcome associated with women after AAA surgery.

## Methods

### Study design and data source

A retrospective study from the Dutch Surgical Aneurysm Audit (DSAA) was conducted in accordance with the STROBE statement^13^. The DSAA is a nationwide and mandatory quality registry that was initiated in 2013, and obtains data on all patients who undergo surgery for an aortic aneurysm in the Netherlands across 61 hospitals.

### Study population

Patients eligible for the present study were women and men registered in the DSAA who underwent primary elective OSR or EVAR for an asymptomatic AAA between January 2013 and December 2018.

### Variables and definitions

Patient- and hospital-related factors considered to have an impact on mortality from a clinical point of view and/or known from the literature were assessed before the analysis by means of a directed acyclic graph to minimize bias (*Table S1*). The patient-related risk factors age, AAA diameter, cardiac and pulmonary co-morbidity, serum creatinine levels, and type of repair were extracted from the registry. Cardiac and pulmonary co-morbidities were registered in the DSAA in accordance with POSSUM^14^. This score is used to predict 30-day mortality and morbidity rates after surgery, and was designed specifically for surgical audit purposes^15,16^. The hospital-related factor hospital volume was divided into OSR and EVAR volume, as the separate volumes can be differently associated with mortality^17^. The volumes of both types of repair were calculated as the total number of primary elective repairs in each hospital throughout the 6-year study period. The total number of patients who had surgery for an aortic aneurysm per hospital was used to calculate hospital volume, regardless of whether patients had missing values on patient-related risk factors as all of the registered repairs add to the cumulative hospital expertise.

### Outcome

The primary outcome of interest for this study was the effect of sex on perioperative mortality, comprising in-hospital mortality during primary admission and 30-day mortality.

### Statistical analysis

Baseline characteristics of both the total study cohort and complete cases are reported, along with hospital characteristics including the percentage of women treated with OSR and EVAR per hospital. Continuous variables, stratified by sex, are reported as mean (s.d.) or median (i.q.r.), depending on the distribution. Categorical variables are reported as absolute numbers with percentages.

The data can be regarded to have a clustered structure as they were obtained from 61 hospitals, whereby patients from the same hospital formed a single cluster (group). It is possible that patients treated in the same hospital have correlated outcomes^18,19^. A wide variety of factors may lead to higher or lower mortality rates in particular hospitals. To deal with possible correlated outcomes, a mixed-effects logistic regression model was used. The following were used as fixed effects in the analysis: OSR volume, EVAR volume, age, sex, AAA diameter, cardiac and pulmonary co-morbidity, serum creatinine levels, and type of repair. The random effect in the statistical model was a hospital-specific offset, which was assumed to follow a normal distribution with a mean of zero. To assess the degree of correlation between patients treated in the same hospital, the intraclass correlation coefficient (ICC) was calculated^19^. The ICC is calculated by dividing the random-effect variance (between-hospital variance) by the total unexplained variance (between-hospital variance and assumed within-hospital variance; fixed value of π^2^/3 in standard logistic distribution)^19,20^.

The analysis included patients with complete data on patient-related risk factors (complete-case analysis). The association between variables and perioperative mortality was expressed as odds ratios (ORs) with the corresponding 95 per cent confidence intervals. *P*<0.050 was considered statistically significant. Statistical analyses were conducted with SPSS^®^ 25 (IBM, Armonk, NY, USA) and R studio version 1.3.959 (The R Foundation for Statistical Computing, Vienna, Austria).

## Results

### Participants and descriptive data

**Fig. 1:**
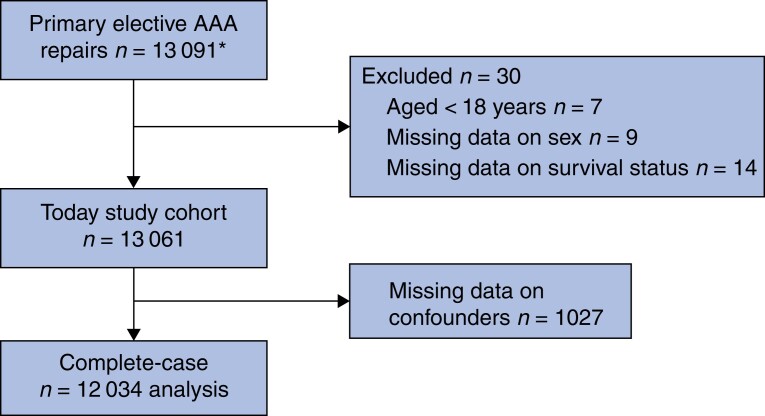
Study flow diagram

Some 13 091 patients who underwent elective primary AAA repair in 61 hospitals were registered in the DSAA (*Fig. 1*). These data were used to calculate hospital volume. After exclusion of 30 patients aged 18 years or under, or for whom information on sex or mortality was missing, the total study cohort comprised 13 061 patients. Data were considered to be missing completely at random as patients with missing values on patient-related factors were not treated at specific hospitals. Hence, no hospital was excluded from the analysis. Ultimately, 12 034 patients were included in the complete case-analysis. A total of 2827 patients were treated with OSR (550 women, 19.5 per cent) and 9207 with EVAR (1230 women, 13.4 per cent). Women were older than men at the time of surgery: mean(s.d.) 74.1(7.7) *versus* 73.1(7.6) years respectively (*P* < 0.001) (*Table 1*).

*Used in calculation of hospital volume. AAA, abdominal aortic aneurysm.

**Table 1:** Baseline characteristics of patients undergoing primary elective abdominal aortic aneurysm repair in the Netherlands (2013–2018)

	Total study cohort		Complete cases	
	Women (*n* = 1916)	Men (*n* = 11 145)	Women (*n* = 1780)	Men (*n* = 10 254)
Age (years)*	74.1 (7.7)	73.1 (7.6)	74.1 (7.7)	73.1 (7.6)
**AAA diameter (mm)**†	55 (52–60)	58 (55–65)	55 (52–60)	58 (55–65)
Missing	30 (1.6)	125 (1.1)		
**Preoperative cardiac status**				
No cardiac history	820 (43.9)	4702 (43.7)	780 (43.8)	4492 (43.8)
Medication for hypertension, angina pectoris, diuretics or digoxin	883 (47.3)	4889 (45.4)	841 (47.2)	4631 (45.2)
Peripheral oedema, anticoagulation (vitamin K antagonist), borderline cardiomyopathy	144 (7.7)	995 (9.2)	138 (7.8)	957 (9.3)
Increased central venous pressure, cardiomegaly	21 (1.1)	182 (1.7)	21 (1.2)	174 (1.7)
Missing	48 (2.5)	377 (3.4)		
**Preoperative pulmonary status**				
No dyspnoea	1313 (69.6)	8280 (75.4)	1232 (69.2)	7715 (75.2)
Dyspnoea on exertion	476 (25.2)	2281 (20.8)	457 (25.7)	2145 (20.9)
Disabling dyspnoea	72 (3.8)	318 (2.9)	69 (3.9)	302 (2.9)
Dyspnoea at rest, consolidation, fibrosis	26 (1.4)	100 (0.9)	22 (1.2)	92 (0.90)
Missing	29 (1.5)	166 (1.5)		
**Creatinine (µmol/l)**†	76 (65–92)	92 (79–110)	76 (64–92)	92 (79–109)
Missing	44 (2.3)	305 (2.7)		

Values in parentheses are percentages unless indicated otherwise; values are *mean (s.d.) and †median (i.q.r.). Baseline characteristics are shown for 13 061 patients who underwent primary elective abdominal aortic aneurysm (AAA) repair after exclusion of those aged 18 years or less, or with missing values on sex or mortality, and for 12 034 patients after excluding those whose data set was incomplete.

### Hospital characteristics

The median total number of elective AAA repairs over 6 years was 243 (i.q.r. 187–320) per hospital. The median OSR volume was 55 (39–78) and median EVAR volume was 193 (140–240). Some 18.8 per cent of all patients treated by OSR per hospital, and 12.9 per cent of all those treated by EVAR per hospital, were women (*Table 2*).

**Table 2:** Type of repair and proportion of women treated per hospital across 61 hospitals in the Netherlands (2013–2018)

	Total study cohort (*n* = 13 061)	Complete cases (*n* = 12 034)
**% OSR per hospital**	23.2 (16.5–30.0)	23.1 (16.3–29.1)
**% women treated per hospital**	14.8 (12.6–16.2)	14.6 (12.7–16.0)
**% women treated by OSR per hospital**	18.2 (14.7–21.8)	18.8 (15.2–22.9)
**% women treated by EVAR per hospital**	12.6 (10.5–15.2)	12.9 (10.7–15.4)

Values are median (interquartile range). OSR, open surgical repair. EVAR, endovascular aneurysm repair.

### Mortality data

The overall mortality rate was higher in women than men (53 of 1780 (3.0 per cent) *versus* 152 of 10 254 (1.5 per cent); *P* < 0.001). Mortality rates were higher in women than in men after both OSR (38 of 550 (6.9 per cent) *versus* 104 of 2277 (4.6 per cent); *P* = 0.024) and EVAR (15 of 1230 (1.2 per cent) *versus* 48 of 7977 (0.6 per cent); *P* = 0.014).

### Mixed-effects logistic regression analysis

After adjusting for patient- and hospital-related factors, female sex was significantly associated with perioperative mortality (OR 1.68, 95 per cent c.i. 1.20 to 2.37). Advanced age, cardiac and pulmonary co-morbidity, higher serum creatinine levels, and OSR as type of repair were also associated with an increased mortality risk (*Table 3*). Higher hospital OSR volume was associated with a lower risk of mortality (OR 0.91 (0.85 to 0.95) per 10-procedure increase), whereas higher hospital EVAR volume was associated with a higher risk of death (OR 1.03 (1.01 to 1.05) per 10-procedure increase).

**Table 3:** Mixed-effects logistic regression analysis to determine the effect of sex on 30-day and/or in-hospital mortality following elective abdominal aortic aneurysm repair in the Netherlands

	Odds ratio	*P*
**Sex (F *versus* M)**	1.68 (1.20, 2.37)	0.003
**Age (per year)**	1.07 (1.05, 1.10)	< 0.001
**AAA diameter (per 10 mm)**	1.04 (0.91, 1.18)	0.577
**Cardiac co-morbidity**		
No cardiac history	1.00 (reference)	
Medication for hypertension, angina pectoris, diuretics or digoxin	1.31 (0.94, 1.83)	0.108
Peripheral oedema, anticoagulation (vitamin K antagonist), borderline cardiomyopathy	1.86 (1.17, 2.95)	0.009
Increased central venous pressure, cardiomegaly	2.64 (1.12, 6.23)	0.026
**Pulmonary co-morbidity**		
No dyspnoea	1.00 (reference)	
Dyspnoea on exertion	2.36 (1.73, 3.21)	< 0.001
Disabling dyspnoea	2.12 (1.03, 4.36)	0.042
Dyspnoea at rest, consolidation, fibrosis	8.33 (3.86, 17.99)	< 0.001
**Creatinine (per 100-µmol/l increase)**	1.61 (1.33, 1.96)	< 0.001
**Type of repair (OSR *versus* EVAR)**	12.23 (8.69, 17.23)	< 0.001
**Hospital volume OSR (per 10 procedures)**	0.91 (0.85, 0.95)	0.002
**Hospital volume EVAR (per 10 procedures)**	1.03 (1.01, 1.05)	0.017

Values in parentheses are 95 per cent confidence intervals. OSR, open surgical repair; EVAR, endovascular aneurysm repair. These are the results of the analysis investigating the effect of sex on perioperative mortality, with correction for confounders.

The estimated hospital-specific offset variance across hospitals was 0.08. An ICC of 0.024 (2.4 per cent) suggested that the outcomes of patients treated in the same hospital were only slightly correlated.

## Discussion

The associations between both patient- and hospital-related factors and mortality in AAA surgery have been well reported previously. In the investigation of the higher mortality rate in women following elective AAA repair, contemporary studies^2,3,5^ have focused foremost on patient-related risk factors. As hospital-level factors can affect outcomes as well, a study combining both factors was conducted to find an explanation beyond patient-related factors for why women are at higher risk. Using nationwide data on aortic aneurysm repair in the Netherlands, the present study found that female sex was associated with mortality after additional correction for interhospital variation.

These findings corroborated those of a recent study^21^ that investigated sex as a modifier in the volume–outcome relationship. The authors concluded that female sex was associated with increased mortality and that hospital volume did not have a consistent effect in women^21^. Another study^11^ that investigated various hospital-level variables showed that institutional practice patterns had a relatively minor impact on mortality in comparison to patient-level risk factors. These reports suggest that factors at patient level may be more important in explaining the higher mortality risk among women. The patient-level risk factors advanced age, cardiac and pulmonary co-morbidity, high serum creatinine levels, and OSR as type of repair were associated with mortality in the present study, in agreement with previous studies^6,22,23^. Further in-depth research on other patient-related risk factors, such as anatomical, genetic or biological differences between women and men, are needed to identify potential explanations for the sex-specific mortality risk.

Hospital volume as a measurable parameter at hospital level was used as a proxy to express possible variation in expertise in AAA surgery and other hospital-related processes, such as resources for dealing with postoperative complications. Cumulative number of OSR or EVAR procedures performed over 6 years in each hospital was used as hospital volume, which can be considered to correspond to the average annual volume used in previous studies^24–26^. The focus of the present analysis was the mortality risk among patients who underwent elective repair. As such, ruptured AAA procedures were not taken into the calculation of hospital volume, which may not have done justice to tertiary referral centres that performed more repairs for ruptured AAA than other centres and may potentially have affected outcomes. Although different definitions of volume have been used, higher hospital volume is reported to be associated with lower mortality after AAA repair^27–29^. However, there seems to be a difference in strength of the association of OSR and EVAR volume with mortality. Previous research^17^ has shown that the association between OSR volume and mortality is stronger than that for EVAR volume. Although higher OSR volume was associated with lower mortality as identified previously^24,30^, EVAR volume was associated with a slightly higher mortality risk in the present study. As EVAR has a relatively low mortality rate and EVAR volume has been reported to have no or little relationship with mortality, a possible explanation for this surprising observation is that selection bias had occurred^24,25^. EVAR as a less invasive operation is often the procedure of choice in older patients with more co-morbidity, and/or offered to a broader selection of patients^31–34^. Alternatively, heterogeneity in definitions of hospital volume may also have had an impact on differences in outcomes. For example, some analyses^4–6^ used the average annual hospital volume, whereas another study^35^ used the annual hospital volume. Although these studies revealed similar outcomes for OSR (volume–outcome association), a minor difference was noted for EVAR (minor volume–outcome association or no association). Notably, the interpretation of the volume–outcome relationship in the present study is different from that in studies that investigated the effect of hospital volume on the mortality risk of patients undergoing OSR or EVAR^25,26,35^. As such, the hospital volume–outcome relationship can be investigated in various ways, reflecting the complexity of the underlying mechanisms.

The study aimed to control for possible unexplained interhospital variation that may have affected patient outcomes, by accounting for possible correlation between outcomes of patients treated in the same hospital. Hospital parameters that may vary included the concept of heterogeneity in differences in surgical experience with type of repair or differences in experience with postoperative AAA care in women. As a secondary finding, the analysis showed that there was no heterogeneity between hospitals after correction for the fixed effects; all hospitals performed equally. Although the study aimed to capture these unmeasured hospital-related parameters, there is a possibility that the authors could not have accounted for all such factors.

The study showed that female sex is associated with high mortality after elective AAA repair. The high mortality risk in women may in part be due to a minor delay in treatment, reflected by a median AAA diameter of 5.5 cm, with potentially more advanced AAA disease and need for complex repair. However, as women are at higher risk of perioperative mortality, perhaps the trade-off of treating women with surgery should be re-evaluated. The threshold for treating AAA in women is currently set at an aortic diameter of 5.0 cm, which is lower than the threshold of 5.5 cm in men, possibly because women have a higher risk of aneurysm rupture than men^1,36^. Yet, perhaps the perioperative mortality risk exceeds the rupture risk at the lower AAA diameter threshold. As further studies are warranted to investigate this trade-off, a more dynamic approach to treatment may be suggested meanwhile. For women undergoing open repair, the threshold should perhaps be increased until as-yet unidentified risk factors for mortality have been elucidated, whereas a lower threshold may be indicated for EVAR considering the low mortality risk. It is clear that a tailormade decision is required, by incorporating the patient’s preference into shared decision-making^37^.

There were some limitations to this study. First, potential risk factors that were not registered in the DSAA could not be taken into account. These include both patient- and hospital-related factors; the former include AAA parameters such as aneurysm anatomy and operative complexity, and the latter surgeon volume (number of procedures performed per surgeon) which has been proposed to be associated with mortality^38,39^. Social factors such as caregiver status may also influence outcomes, which could not be taken into account in the present analysis. Second, this retrospective study used data from a quality registry that was not primarily designed for research and could have missing values. The percentage of missing values for each co-morbidity was less than 4 per cent and the incomplete data were distributed over approximately 8 per cent of the patients. The information bias of the extracted variables was therefore considered to be acceptable.

## Collaborators

P.J. van den Akker, G.J. Akkersdijk, G.P. Akkersdijk, W.L. Akkersdijk, M.G. van Andringa de Kempenaer, C.H.P. Arts, J.A.M. Avontuur, O.J. Bakker, R. Balm, W.B. Barendregt, J.A. Bekken, M.H. Bender, B.L. Bendermacher, M. van den Berg, P. Berger, R.J. Beuk, J.D. Blankensteijn, R.J. Bleker, J.J. Blok, A.S. Bode, M.E. Bodegom, K.E. van der Bogt, A.P.M. Boll, M.H. Booster, B.L. Borger van der Burg, G.J. de Borst, W.T.G.J. Bos- van Rossum, J. Bosma, J.M.J. Botman, L.H. Bouwman, V. Brehm, M.T. de Bruijn, J.L. de Bruin, P. Brummel, J.P. van Brussel, S.E. Buijk, M.A. Buijs, M.G. Buimer, D.H. Burger, H.C. Buscher, E. Cancrinus, P.H. Castenmiller, G. Cazander, A.M. Coester, P.H. Cuypers, J.H. Daemen, I. Dawson, J.E. Dierikx, M.L. Dijkstra, J. Diks, M.K. Dinkelman, M. Dirven, D.E. Dolmans, R.C. van Doorn, L.M. van Dortmont, J.W. Drouven, M.M. van der Eb, D. Eefting, G.J. van Eijck, J.W. Elshof, B.H. Elsman, A. van der Elst, M.I. van Engeland, R.G. van Eps, M.J. Faber, W.M. de Fijter, B. Fioole, T.M. Fokkema, F.A. Frans, W.M. Fritschy, P.H. Fung Kon Jin, R.H. Geelkerken, W.B. van Gent, G.J. Glade, B. Govaert, R.P. Groenendijk, H.G. de Groot, R.F. van den Haak, E.F. de Haan, G.F. Hajer, J.F. Hamming, E.S. van Hattum, C.E. Hazenberg, P.P. Hedeman Joosten, J.N. Helleman, L.G. van der Hem, J.M. Hendriks, J.A. van Herwaarden, J.M. Heyligers, J.W. Hinnen, R.J. Hissink, G.H. Ho, P.T. den Hoed, M.T. Hoedt, F. van Hoek, R. Hoencamp, W.H. Hoffmann, W. Hogendoorn, A.W. Hoksbergen, E.J. Hollander, M. Hommes, C.J. Hopmans, L.C. Huisman, R.G. Hulsebos, K.M. Huntjens, M.M. Idu, M.J. Jacobs, M.F. van der Jagt, J.R. Jansbeken, R.J. Janssen, H.H. Jiang, S.C. de Jong, T.A. Jongbloed-Winkel, V. Jongkind, M.R. Kapma, B.P. Keller, A. Khodadade Jahrome, J.K. Kievit, P.L. Klemm, P. Klinkert, N.A. Koedam, M.J. Koelemaij, J.L. Kolkert, G.G. Koning, O.H. Koning, R. Konings, A.G. Krasznai, R.M. Krol, R.H. Kropman, R.R. Kruse, L. van der Laan, M.J. van der Laan, J.H. van Laanen, G.W. van Lammeren, D.A. Lamprou, J.H. Lardenoye, G.J. Lauret, B.J. Leenders, D.A. Legemate, V.J. Leijdekkers, M.S. Lemson, M.M. Lensvelt, M.A. Lijkwan, R.C. Lind, F.T. van der Linden, P.F. Liqui Lung, M.J. Loos, M.C. Loubert, K.M. van de Luijtgaarden, D.E. Mahmoud, C.G. Manshanden, E.C. Mattens, R. Meerwaldt, B.M. Mees, G.C. von Meijenfeldt, T.P. Menting, R. Metz, R.C. Minnee, J.C. de Mol van Otterloo, M.J. Molegraaf, Y.C. Montauban van Swijndregt, M.J. Morak, R.H. van de Mortel, W. Mulder, S.K. Nagesser, C.C. Naves, J.H. Nederhoed, A.M. Nevenzel-Putters, A.J. de Nie, D.H. Nieuwenhuis, J. Nieuwenhuizen, R.C. van Nieuwenhuizen, D. Nio, V.J. Noyez, A.P. Oomen, B.I. Oranen, J. Oskam, H.W. Palamba, A.G. Peppelenbosch, A.S. van Petersen, B.J. Petri, M.E. Pierie, A.J. Ploeg, R.A. Pol, E.D. Ponfoort, I.C. Post, P.P. Poyck, A. Prent, S. ten Raa, J.T. Raymakers, M. Reichart, B.L. Reichmann, M.M. Reijnen, J.A. de Ridder, A. Rijbroek, M.J. van Rijn, R.A. de Roo, E.V. Rouwet, B.R. Saleem, P.B. Salemans, M.R. van Sambeek, M.G. Samyn, H.P. van ’t Sant, J. van Schaik, P.M. van Schaik, D.M. Scharn, M.R. Scheltinga, A. Schepers, P.M. Schlejen, F.J. Schlosser, F.P. Schol, V.P. Scholtes, O. Schouten, M.A. Schreve, G.W. Schurink, C.J. Sikkink, A. te Slaa, H.J. Smeets, L. Smeets, R.R. Smeets, A.A. de Smet, P.C. Smit, T.M. Smits, M.G. Snoeijs, A.O. Sondakh, M.J. Speijers, T.J. van der Steenhoven, S.M. van Sterkenburg, D.A. Stigter, R.A. Stokmans, R.P. Strating, G.N. Stultiëns, J.E. Sybrandy, J.A. Teijink, B.J. Telgenkamp, M. Teraa, M.J. Testroote, T. Tha-In, R.M. The, W.J. Thijsse, I. Thomassen, I.F. Tielliu, R.B. van Tongeren, R.J. Toorop, E. Tournoij, M. Truijers, K. Türkcan, R.P. Tutein Nolthenius, Ç. Ünlü, R.H. Vaes, A.A. Vafi, A.C. Vahl, E.J. Veen, H.T. Veger, M.G. Veldman, S. Velthuis, H.J. Verhagen, B.A. Verhoeven, C.F. Vermeulen, E.G. Vermeulen, B.P. Vierhout, R.J. van der Vijver-Coppen, M.J. Visser, J.A. van der Vliet, C.J. Vlijmen - van Keulen, R. Voorhoeve, J.R. van der Vorst, A.W. Vos, B. de Vos, C.G. Vos, G.A. Vos, M.T. Voute, B.H. Vriens, P.W. Vriens, A.C. de Vries, D.K. de Vries, J.P. de Vries, M. de Vries, C. van der Waal, E.J. Waasdorp, B.M. Wallis de Vries, L.A. van Walraven, J.L. van Wanroij, M.C. Warlé, W. van de Water, V. van Weel, A.M. van Well, G.M. Welten, R.J. Welten, J.J. Wever, A.M. Wiersema, O.R. Wikkeling, W.I. Willaert, J. Wille, M.C. Willems, E.M. Willigendael, E.D. Wilschut, W. Wisselink, M.E. Witte, C.H. Wittens, C.Y. Wong, R. Wouda, O. Yazar, K.K. Yeung, C.J. Zeebregts, M.L. van Zeeland.

## Supplementary Material

znab465_Supplementary_Data
