# Supplementary material for: Mortality following elective abdominal aortic aneurysm repair in women
Source: Br J Surg. 2022 Mar 3;109(4):340–5. doi: 10.1093/bjs/znab465 (PMC10364697; doi:10.1093/bjs/znab465)
Supplement: znab465_Supplementary_Data [file znab465_supplementary_data.docx]

**Table S1.** Directed Acyclic Graph


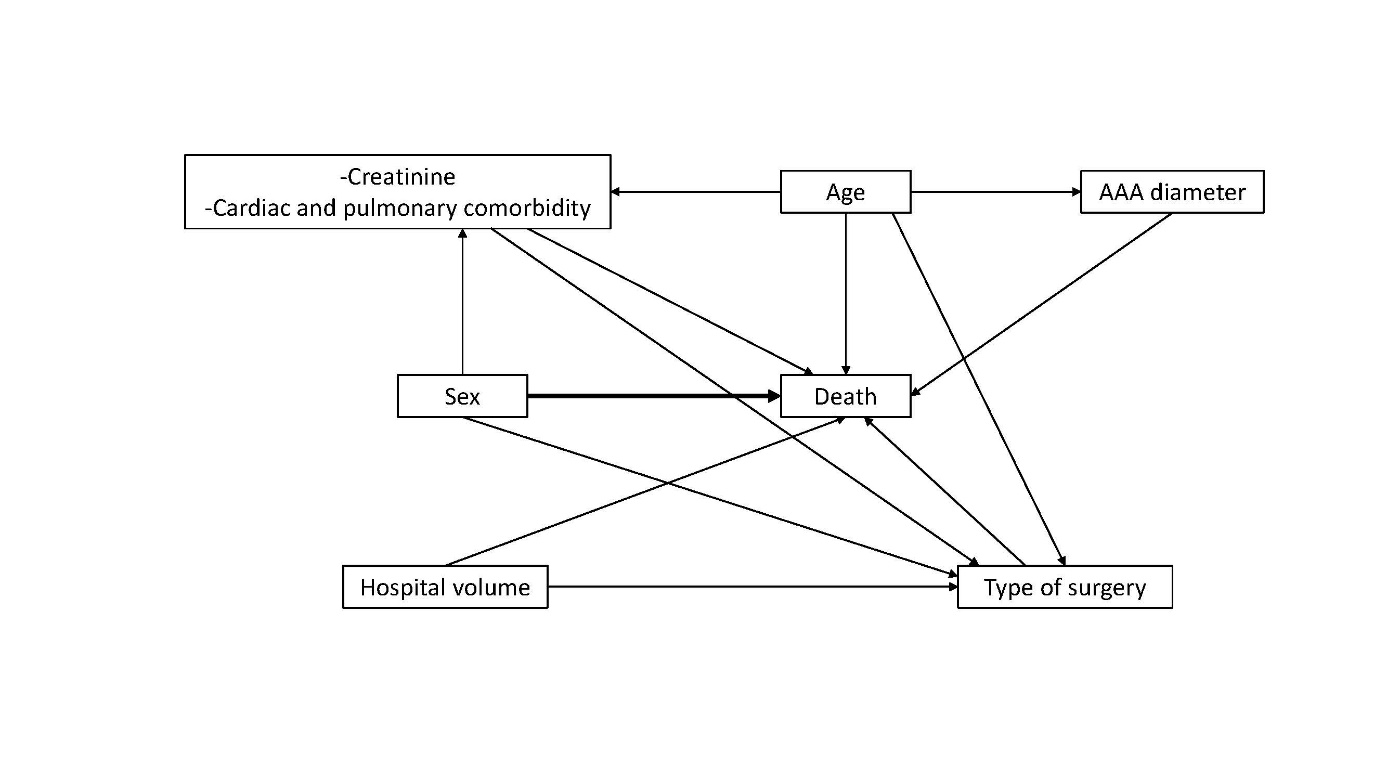


Display of causal assumptions between the variables. The primary interest is the effect of sex on death. Creatinine, cardiac and pulmonary comorbidity, age, AAA diameter, type of surgery and hospital volume were confounders in the statistical model.
